# Luteolin intake is negatively associated with all-cause and cardiac mortality among patients with type 2 diabetes mellitus

**DOI:** 10.1186/s13098-023-01026-9

**Published:** 2023-03-25

**Authors:** Wenbin Zhang, Duanbin Li, Yu Shan, Yecheng Tao, Qingqing Chen, Tianli Hu, Menghan Gao, Zhezhe Chen, Hangpan Jiang, Changqin Du, Min Wang, Kai Guo

**Affiliations:** 1 Department of Cardiology, Sir Run Run Shaw Hospital, College of Medicine, Zhejiang University, Hangzhou, Zhejiang People’s Republic of China; 2 Key Laboratory of Cardiovascular Intervention and Regenerative Medicine of Zhejiang Province, Hangzhou, Zhejiang People’s Republic of China; 3 Department of Cardiology, Xinhua Hospital, Shanghai Jiao Tong University School of Medicine, 1665 Kongjiang Road, Shanghai, 200092 People’s Republic of China; 4 Department of Cardiology, Zhejiang Hospital, Hangzhou, Zhejiang People’s Republic of China; 5 Department of Cardiology, The Fourth Affiliated Hospital, College of Medicine, Zhejiang University, Yiwu, Zhejiang People’s Republic of China; 6 College of Medicine, Zhejiang University, Hangzhou, Zhejiang People’s Republic of China

**Keywords:** Luteolin, All-cause mortality, Cardiac mortality, Diabetes, NHANES

## Abstract

**Background:**

Luteolin, a common flavonoid in our daily diet, has potent anti-diabetic effects. However, its prognostic impact on type 2 diabetes mellitus (T2DM) is still uncertain. This study aimed to clarify this association.

**Methods:**

In this prospective cohort study, 2,461 patients with T2DM were included from the National Health and Nutrition Examination Survey. Dietary luteolin intake was estimated by the type and amount of food consumed in a 24-hour dietary recall. All-cause and cardiac mortality were ascertained by National Death Index Mortality data (as of December 31, 2019). The association of luteolin intake with mortality risk was estimated by Cox proportional hazards model.

**Results:**

The median (interquartile range) luteolin intake was 0.355 (0.130, 0.835) mg/day. During the follow-up (median, 8.4 years), 561 all-cause deaths (including 136 cardiac deaths) were documented. Per-unit increment of luteolin intake (natural logarithm transformed) was found to reduce all-cause mortality by 7.0% (*P* = 0.024) and cardiac mortality by 22.6% (*P* = 0.001) in patients with T2DM. An inverse dose-response association was identified between luteolin intake (range: 0.005–9.870 mg/day) and mortality risk. The consistent result was also shown when stratified by age, gender, race, body mass index, HbA1c level, and T2DM duration. Moreover, luteolin intake increment was also shown to be associated with a lower C-reactive protein level at baseline (β =-0.332; 95% CI =-0.541, -0.122).

**Conclusion:**

The current study confirmed that the dietary luteolin intake increment reduced all-cause mortality (especially cardiac mortality) in patients with T2DM, which may be attributed to the anti-inflammatory property of luteolin.

**Graphical abstract:**

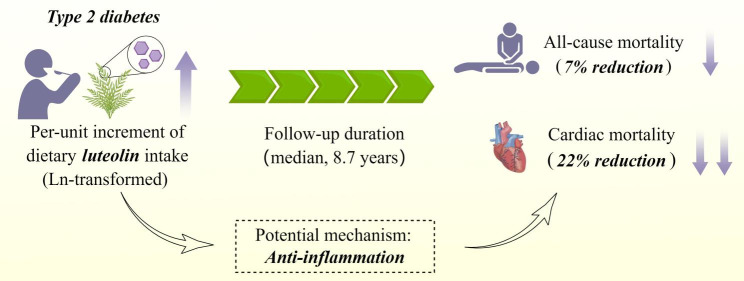

**Supplementary Information:**

The online version contains supplementary material available at 10.1186/s13098-023-01026-9.

## Background

Type 2 diabetes mellitus (T2DM) is a major global public health concern, with a total of 415 million people living with diabetes worldwide [1]. As the sixth leading cause of disability, T2DM carries tremendous healthcare and financial burden [2, 3]. Patients with T2DM have substantially higher cardiovascular disease morbidity and mortality [4]. The World Health Organization reported that cardiovascular disease accounted for more than two-thirds of diabetes-related deaths in the elderly [5]. For mechanisms, T2DM accelerates most cardiac pathologies, including microvascular dysfunction, vascular endothelial inflammation and injury, thrombogenesis, and autonomic nerve disorder [6]. As a metabolic disease, T2DM is inevitably related to our daily diet. In the United States, almost half of cardiovascular and diabetes deaths are linked to poor dietary practices, corresponding to nearly 1,000 deaths per day [7]. Therefore, it is important to identify a healthy dietary pattern to avoid premature cardiovascular complications and mortality in patients with T2DM.

Luteolin (molecular formula C_15_H_10_O_6_), a naturally occurring metabolite, is commonly found in our dietary components, such as parsley, thyme, peppermint, basil, celery, and artichoke [8]. Among the thousands of flavonoids, luteolin belongs to the flavones class and is often present in the form of its glycoside (luteolin-7-O-glucoside or luteolin-7G) [9, 10]. The health-promoting benefits of luteolin have been well documented due to its pleiotropic properties, including anti-diabetic [11], cardiovascular protective [12], anti-inflammatory [13], and anti-cancer [14] effects. In diabetic animal models, luteolin has been identified to reverse glucose intolerance [15], delay renal function decline [16], improve learning and memory [17], and promote wound healing [18]. Moreover, in various animal models of cardiac disease, the therapeutic effects of luteolin were also shown, including promoting cytoprotection in myocardial ischemia/reperfusion injury [19], improving heart function in heart failure [20], and reversing atherosclerosis in coronary artery disease [21]. Overall, the potent antidiabetic and cardiovascular protective effects of luteolin have been extensively documented in these animal studies. However, there are few population-based studies to investigate its relationship with relevant clinical outcomes.

Therefore, we designed this population-based cohort study to investigate the impact of dietary luteolin intake on the prognosis of T2DM patients, including all-cause and cardiac mortality.

## Methods

### Study population

This study employed the data from the National Health and Nutrition Examination Survey (NHANES) (3 cycles of 2007–2008, 2009–2010, and 2017–2018). NHANES was sponsored and administered by the National Center for Health Statistics, which investigated the US civilians’ health and nutritional status through a nationally representative sample [22]. Participants were required to receive a series of questionnaires, physical examinations, and laboratory tests at home or the mobile examination center (MEC). More details were presented elsewhere [22].

Overall, 3,489 diabetic patients were identified according to diabetic questionnaires (variable diq010), blood glucose testing in MEC (variables lbxgh, lbdglusi, lbdsglsi, lbdgltsi), and records of anti-diabetic agents use (variable rxddcn). Furthermore, we excluded those with luteolin intake missing (n = 623), sample weights missing (n = 196), follow-up data missing (n = 64), underlying type 1 diabetes (told to have diabetes when < 30 years, n = 145) [23], and pregnant (n = 0). Finally, 2,461 patients with T2DM were enrolled from NHANES (Figure S1).

### Dietary intakes

NHANES collected the amount of food and beverages consumed by participants in the past 24 h. Two 24-hour dietary recall interviews were performed, including an in-person interview in MEC and a telephone interview 3–10 days afterward. The specific nutrient was further calculated according to the Food and Nutrient Database for Dietary Studies (FNDDS) [24]. These nutrient intakes were averaged if participants underwent both the first and second dietary recall interviews. We extracted the intake data on 29 types of flavonoids in 6 categories (including luteolin). The dictionary of luteolin content in foods and beverages (≥ 1 mg/100 mg) was shown in Table S1.

### Mortality outcomes

NHANES Public-Use Linked Mortality Files were used to determine the survival status of participants (as of December 31, 2019) [25]. The International Classification of Diseases, Tenth Revision (ICD-10) was used to define the cause-specific death [26]. We examined the all-cause death and the top four cause-specific death, in order of cardiac diseases (ICD-10: I00-I09, I11, I13, I20-I51), malignant neoplasms (ICD-10: C00-C97), diabetes mellitus (ICD-10: E10-E14), and cerebrovascular diseases (ICD-10: I60-I69). The definitions and proportions of cause-specific death were shown in Table S2.

### Covariate definitions

Demographic parameters were extracted from the questionnaire data. Races were classified into non-Hispanic whites, non-Hispanic blacks, and others. Alcohol consumption in the past 12 months was defined as heavy drinking (≥ 2 drinks/day), mild drinking (1 drink/day), and non-drinking (no drink). Cigarette consumption was defined as never smoked (< 100 cigarettes in a lifetime), formerly smoked (≥ 100 cigarettes in a lifetime and quit now), and currently smoked (≥ 100 cigarettes in a lifetime and smoke some days or every day). Physical activity in leisure time was categorized into no or unable to activity, moderate activity, and vigorous activity. The family poverty income ratio is equal to the family income divided by the poverty guideline, which is corresponding to the year and the state of the participants. Body mass index (BMI, kg/m^2^) is equal to weight (kg) divided by height (m) squared.

Hypertension was defined when diastolic blood pressure ≥ 90 mmHg, systolic blood pressure ≥ 140 mmHg, use of anti-hypertensive drugs (bpq040a), or “yes” code for “told you had high blood pressure” (variable bpq020/030). Hyperlipidemia was defined as current use of lipid-lowering agents or the presence of abnormal lipid profiles, including total cholesterol ≥ 200 mg/dL, triglycerides ≥ 150 mg/dL, low-density lipoprotein cholesterol (LDL-C) ≥ 130 mg/dL, or high-density lipoprotein cholesterol < 40 mg/dL for male whereas < 50 mg/dL for female [27].

### Statistical analysis

Due to the complex survey design, sample weights (day one dietary weights) were taken into account in analyses. The continuous variable was presented as weighted mean ± standard error with comparisons by the Kruskal-Wallis test, and the categorical variable was presented as unweighted count (weighted percentage) with comparisons by the chi-square test.

The events per variable (EPV) criterion was used to evaluate the sample size. If the EPV of the model exceeded 10, the sample size was considered sufficient to meet the statistical requirements [28, 29]. The relative risks of all-cause and cardiac mortality were calculated for luteolin intake tertiles. Kaplan-Meier survival analyses and Cox proportional hazards models were employed to process the survival data. Three statistical models were fitted. The first model adjusted for age (continuous), race (non-Hispanic white, non-Hispanic black, or others), and gender (male or female). The second model further adjusted for BMI (< 20, 20–24, 25–29, or ≥ 30 kg/m^2^), educational attainment (below high school, high school, or college or above), alcohol consumption (none, mild, or heavy), cigarette consumption (never, former, or current), poverty income ratio (≤ 1, 1–3, or > 3), energy intake (< 1500 or ≥ 1500 kcal/day), and physical activity in leisure time (no or unable, moderate, or vigorous activity). Moreover, the third model additionally adjusted for hyperlipidemia diagnosis (no or yes), hypertension diagnosis (no or yes), HbA1c level (< 6.5 or ≥ 6.5%), and use of oral anti-diabetic agents or insulin (no or yes). To minimize the removal of samples, missing covariates were treated by multiple imputations.

A dose-response association between continuous luteolin intake (Ln-transformed) and mortality risk was visualized by the restricted cubic spline model. Four knots of the spline model were determined at specific distribution percentiles (5%, 35%, 65%, and 95%). The likelihood ratio test was used to determine the non-linearity of the dose-response association by comparing the model with and without spline terms.

Subgroup analysis was conducted when patients were stratified by age (< 65, ≥ 65 years), gender (male, female), race (non-Hispanic white, others), BMI (< 30, ≥ 30 kg/m^2^), energy intake (< 1500, ≥ 1500 kcal/day), HbA1c level (< 6.5, ≥ 6.5%), and T2DM duration (< 10, ≥ 10 years). Interactions were examined by integrating the product term between continuous luteolin intake and stratified factors.

To test the robustness, we also conducted several sensitivity analyses. First, the flavonoid database provided the amounts of 29 flavonoids (belonging to 6 flavonoid categories) (Table S3). To avoid the potential effect of remaining flavonoids, we further adjusted other flavonoid intakes in the model. Second, given the underlying luteolin intake outliers, we fitted a new dose-response association after excluding luteolin intake outside the 5th and 95th percentiles. Third, we also examined the association of luteolin intake with cause-specific death, including malignant neoplasms (C00-C97), diabetes mellitus (E10-E14), and cerebrovascular diseases (I60-I69). Finally, to investigate the potential mechanism by which luteolin reduces mortality risk, we examined the association between dietary luteolin intake and baseline cardiometabolic risk factors, including HbA1c, fasting blood glucose, homeostasis model assessment-insulin resistance (HOMA-IR), HOMA-insulin sensitivity (HOMA-IS), HOMA-β, arterial blood pressure, LDL-C, C-reactive protein (CRP).

Two-sided *P* values < 0.05 were considered statistical significance. Data were analyzed by R software (R version 4.1.1).

## Results

The characteristics of 2,461 patients with T2DM were summarized according to luteolin intake tertiles (mean age, 61.3 years; male, 49.4%) (Table 1). The median (interquartile range) of luteolin intake was 0.355 (0.130, 0.835) mg/day. Patients with higher luteolin intake were more likely to be male, tended to have higher educational attainment, higher energy intake, and more intense leisure-time physical activity.

**Table 1:** Baseline characteristics based on luteolin intake among patients with type 2 diabetes mellitus

Characteristic	Total	Luteolin intake (mg/day)			*P* value
		Tertile 1	Tertile 2	Tertile 3	
		[0.005,0.195)	[0.195,0.640)	[0.640,9.870]	
Number of patients	2461	841	803	817	
Age, years	61.28 ± 0.42	60.69 ± 0.74	61.80 ± 0.72	61.36 ± 0.62	0.560
Gender (%)					0.040*
Female	1222 (50.63)	432 (52.54)	410 (54.01)	380 (45.95)	
Male	1239 (49.37)	409 (47.46)	393 (45.99)	437 (54.05)	
Race (%)					0.001*
Non-Hispanic White	971 (63.58)	340 (65.30)	312 (61.43)	319 (63.86)	
Non-Hispanic Black	603 (14.37)	247 (17.98)	207 (15.23)	149 (10.30)	
Others	887 (22.05)	254 (16.72)	284 (23.35)	349 (25.84)	
Educational attainment (%)					< 0.001*
Below high school	407 (9.34)	147 (9.07)	137 (10.61)	123 (8.49)	
High school or equivalent	1002 (40.64)	379 (46.53)	347 (44.87)	276 (31.55)	
College or above	1052 (50.02)	315 (44.41)	319 (44.52)	418 (59.96)	
Body mass index, kg/m^2^	33.34 ± 0.26	33.64 ± 0.37	32.74 ± 0.35	33.58 ± 0.43	0.150
Family income-poverty ratio (%)					0.002*
≤1.0	475 (13.35)	201 (16.73)	167 (13.67)	107 (9.96)	
1.0–3.0	1178 (41.81)	422 (45.01)	384 (42.88)	372 (37.94)	
>3.0	808 (44.84)	218 (38.26)	252 (43.45)	338 (52.10)	
Leisure-time physical activity (%)					< 0.001*
No or unable	1662 (62.90)	619 (70.43)	562 (66.29)	481 (53.04)	
Moderate	621 (28.67)	179 (25.00)	189 (25.38)	253 (34.89)	
Vigorous	178 (8.43)	43 (4.57)	52 (8.33)	83 (12.07)	
Smoking status (%)					0.010*
Never smoker	1210 (49.51)	390 (48.45)	398 (48.77)	422 (51.12)	
Former smoker	872 (35.87)	286 (32.47)	282 (36.20)	304 (38.72)	
Current smoker	379 (14.62)	165 (19.09)	123 (15.03)	91 (10.16)	
Alcohol consumption (%)					< 0.001*
Non-drinker	1235 (44.32)	462 (48.53)	427 (47.67)	346 (37.53)	
Mild drinker	554 (25.14)	138 (18.87)	176 (22.68)	240 (33.04)	
Heavy drinker	672 (30.54)	241 (32.59)	200 (29.65)	231 (29.43)	
Energy intake (%)					0.002*
<1500 kcal/day	971 (34.37)	389 (40.73)	318 (35.82)	264 (27.27)	
≥1500 kcal/day	1490 (65.63)	452 (59.27)	485 (64.18)	553 (72.73)	
Hypertension (%)					0.001*
No	632 (27.66)	193 (20.67)	209 (33.36)	230 (29.15)	
Yes	1829 (72.34)	648 (79.33)	594 (66.64)	587 (70.85)	
Hyperlipidemia (%)					0.130
No	314 (11.05)	109 (12.33)	112 (12.43)	93 (8.66)	
Yes	2147 (88.95)	732 (87.67)	691 (87.57)	724 (91.34)	
HbA1c, %	7.02 ± 0.04	7.02 ± 0.06	6.99 ± 0.07	7.04 ± 0.07	0.880
Fasting blood glucose, mmol/L	8.34 ± 0.09	8.29 ± 0.20	8.21 ± 0.14	8.50 ± 0.14	0.340
Duration of diabetes, years	10.19 ± 0.29	9.78 ± 0.40	10.49 ± 0.50	10.33 ± 0.43	0.480
Use of oral anti-diabetic agents (%)					0.500
No	1084 (46.84)	377 (47.37)	347 (44.27)	360 (48.59)	
Yes	1377 (53.16)	464 (52.63)	456 (55.73)	457 (51.41)	
Use of insulin (%)					0.070
No	2054 (82.79)	684 (80.65)	662 (81.08)	708 (86.26)	
Yes	407 (17.21)	157 (19.35)	141 (18.92)	109 (13.74)	

The continuous variable is presented as weighted mean ± standard error. The categorical variable is presented as unweighted count (weighted percentage)

*P < 0.05

**Fig. 1:**
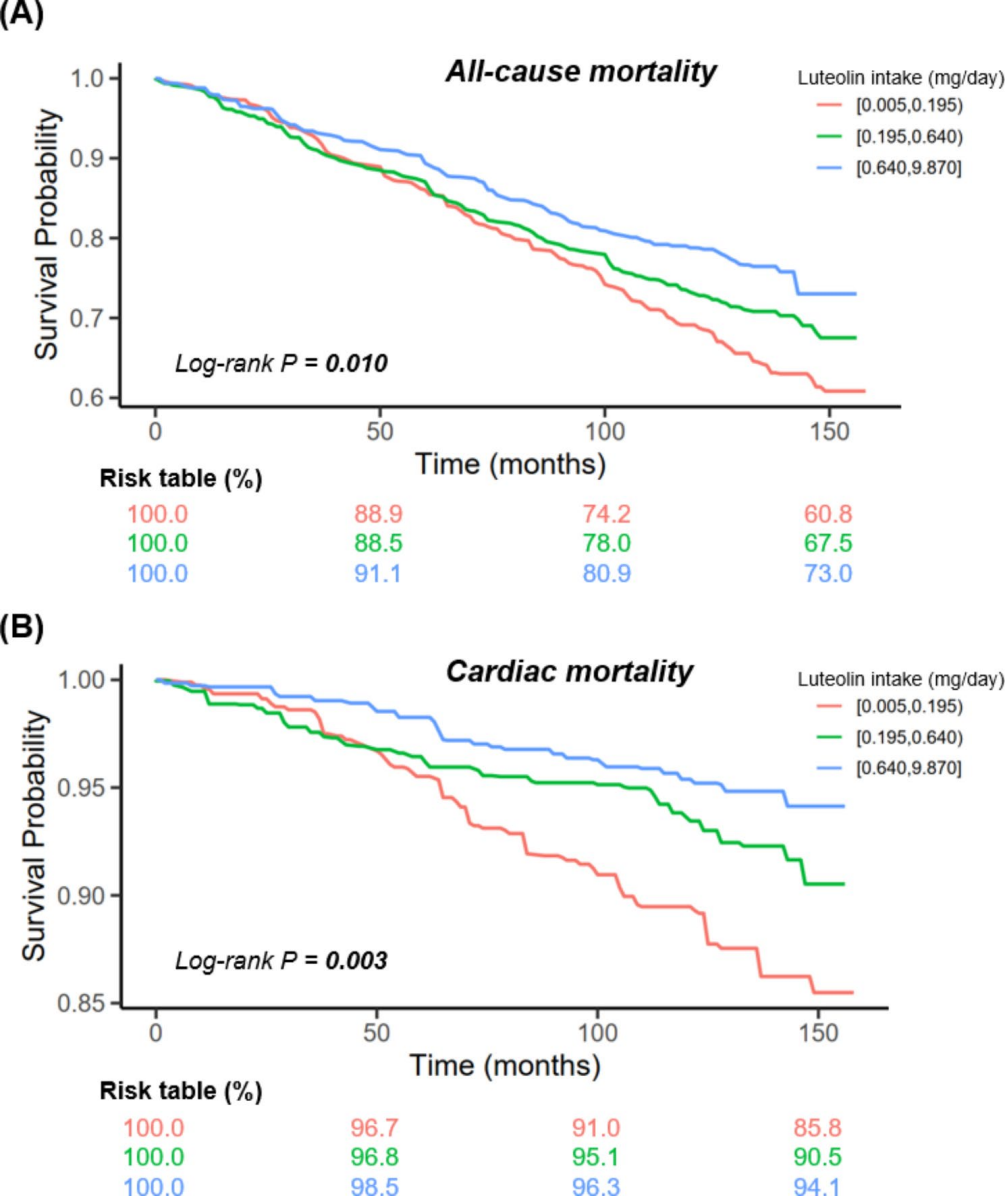
Kaplan-Meier survival curve of all-cause mortality (A) and cardiac mortality (B) based on luteolin intake tertiles among patients with type 2 diabetes mellitus

**Fig. 2:**
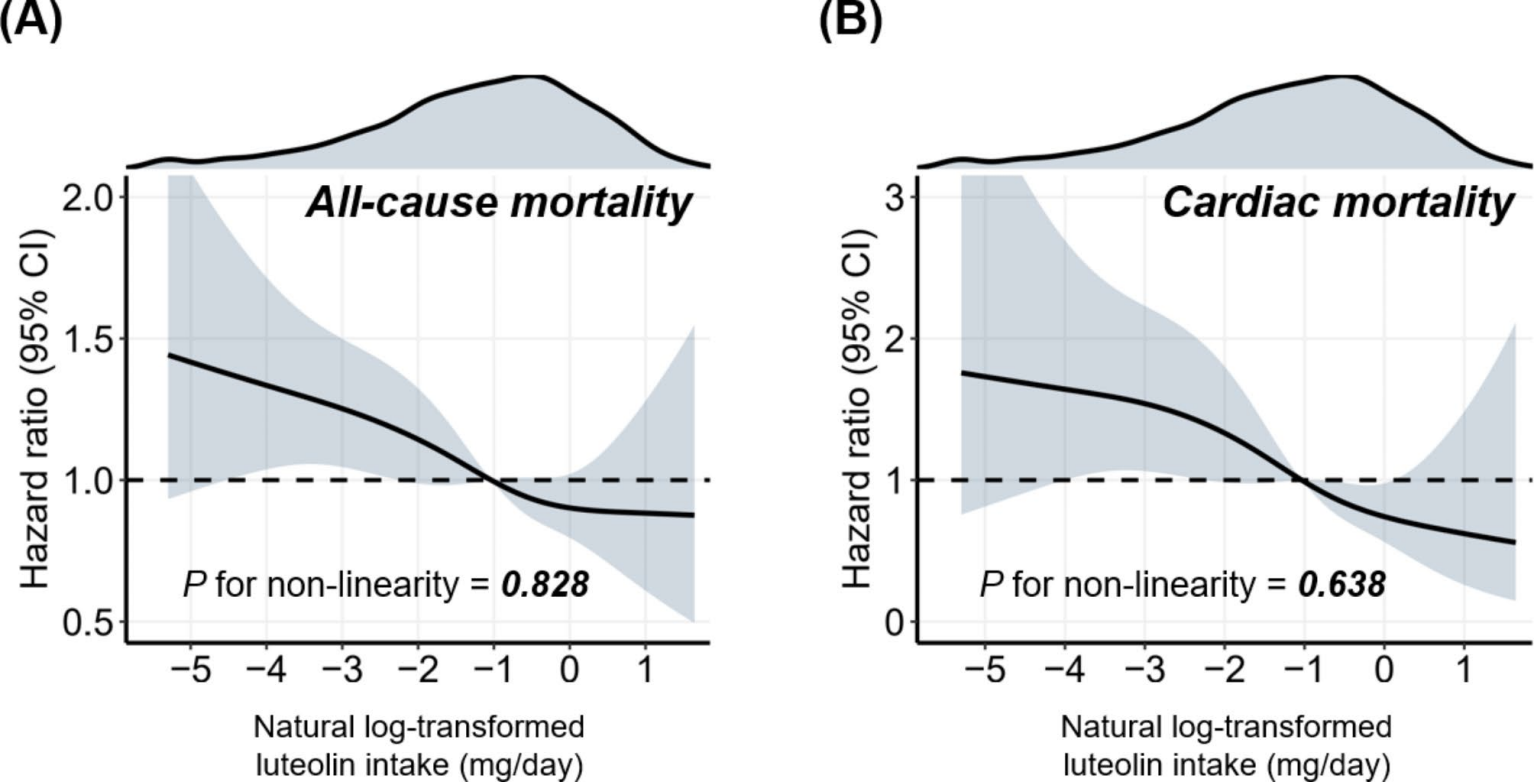
The dose-response association of luteolin intake with all-cause mortality (A) and cardiac mortality (B) among patients with type 2 diabetes mellitus The dose-response association of continuous luteolin intake (Ln-transformed) with mortality risk was visualized by the restricted cubic spline model (corresponding to the range of luteolin intake: 0.005–9.870 mg/day). Four knots of the spline model were determined at specific distribution percentiles (5%, 35%, 65%, and 95%). The spline model was adjusted for consistent confounding factors, including age, gender, race, income-poverty ratio, body mass index, educational attainment, smoking, drinking, energy intake, physical activity, hypertension, hyperlipidemia, HbA1c level, and use of oral anti-diabetic agents or insulin. For more details on confounding factors processing, refer to Table 2. The non-linearity of the dose-response association was examined by the likelihood ratio test. The Y-axis represents the adjusted HR given the value of luteolin intake compared to the corresponding median. The shadow area depicts the 95% confidence intervals. The density diagram at the top depicts the distribution of luteolin intake (Ln-transformed)

During follow-up periods (median, 8.7 years), 561 all-cause deaths (including 136 cardiac deaths) were documented. Given the sufficient positive outcomes, the sample size was considered adequate based on the criterion of EPV greater than 10. Kaplan-Meier survival analyses showed that a higher luteolin intake contributed to a lower all-cause mortality (Log-rank *P* = 0.010) and cardiac mortality (Log-rank *P* = 0.003) (Fig. 1). The relative risks (95% CIs) across luteolin intake tertiles were 1 (reference), 0.910 (0.770, 1.076), and 0.689 (0.573, 0.829) for all-cause mortality and 1 (reference), 0.760 (0.524, 1.102), and 0.481 (0.313,0.740) for cardiac mortality (Table S4). In Cox regression analyses, adjusted-HRs (95% CIs) across luteolin intake tertiles were 1.000 (reference), 0.885 (0.683, 1.146), and 0.855 (0.691, 1.057) for all-cause mortality and 1.000 (reference), 0.662 (0.413, 1.063), and 0.487 (0.284, 0.836) for cardiac mortality (Table 2). Per-unit increment of luteolin intake (Ln-transformed) contributed to a 7.0% reduction in all-cause mortality (adjusted-HR = 0.930; 95% CI = 0.874, 0.991; *P* = 0.024) and a 22.6% reduction in cardiac mortality (adjusted-HR = 0.774; 95% CI = 0.668, 0.898; *P* = 0.001) (Table 2). Moreover, the spline plot showed an inverse dose-response association of luteolin intake (range: 0.005–9.870 mg/day) with all-cause mortality (*P*-nonlinearity = 0.828) and cardiac mortality (*P*-nonlinearity = 0.638) (Fig. 2).

**Table 2:** HR (95% CI) for all-cause and cardiac mortality based on luteolin intake among patients with type 2 diabetes mellitus

Characteristic		Luteolin intake (mg/day)			Per-unit increment of luteolin intake (Ln-transformed)	*P* value
		Tertile 1 [0.005, 0.195)	Tertile 2 [0.195, 0.640)	Tertile 3 [0.640, 9.870]		
All-cause mortality						
	No. deaths/total (%)	221/841 (26.3)	192/803 (23.9)	148/817 (18.1)	561/2461 (22.8)	
	Model 1^1^	1 (reference)	0.866 (0.664, 1.130)	0.668 (0.539, 0.830)	0.864 (0.807, 0.926)	< 0.001
	Model 2^2^	1 (reference)	0.907 (0.701, 1.173)	0.856 (0.690, 1.062)	0.927 (0.869, 0.990)	0.023
	Model 3^3^	1 (reference)	0.885 (0.683, 1.146)	0.855 (0.691, 1.057)	0.930 (0.874, 0.991)	0.024
Cardiac mortality						
	No. deaths/total (%)	62/841 (7.4)	45/803 (5.6)	29/817 (3.5)	136/2461 (5.5)	
	Model 1^1^	1 (reference)	0.649 (0.397, 1.06)	0.385 (0.221, 0.672)	0.725 (0.621, 0.847)	< 0.001
	Model 2^2^	1 (reference)	0.677 (0.408, 1.122)	0.487 (0.279, 0.850)	0.770 (0.659, 0.901)	0.001
	Model 3^3^	1 (reference)	0.662 (0.413, 1.063)	0.487 (0.284, 0.836)	0.774 (0.668, 0.898)	0.001

HR (95% CI) was estimated by Cox proportional hazards model and accounted for the sample weights. Cardiac mortality was defined as I00-I09, I11, I13, I20-I51 according to the ICD-10 criteria

^1^Model 1 was adjusted for age (continuous), race (non-Hispanic white, non-Hispanic black, or others), and gender (male or female)

^2^Model 2 was additionally adjusted for BMI (< 20, 20–24, 25–29, or ≥ 30 kg/m^2^), educational attainment (below high school, high school, or college or above), alcohol consumption (none, mild, or heavy), cigarette consumption (never, former, or current), poverty income ratio (≤ 1, 1–3, or > 3), energy intake (< 1500 or ≥ 1500 kcal/day), and physical activity in leisure time (no or unable, moderate, or vigorous)

^3^Model 3 was additionally adjusted for hyperlipidemia (no or yes), hypertension (no or yes), HbA1c level (< 6.5 or ≥ 6.5%), and use of oral anti-diabetic agents or insulin (no or yes)

**Fig. 3:**
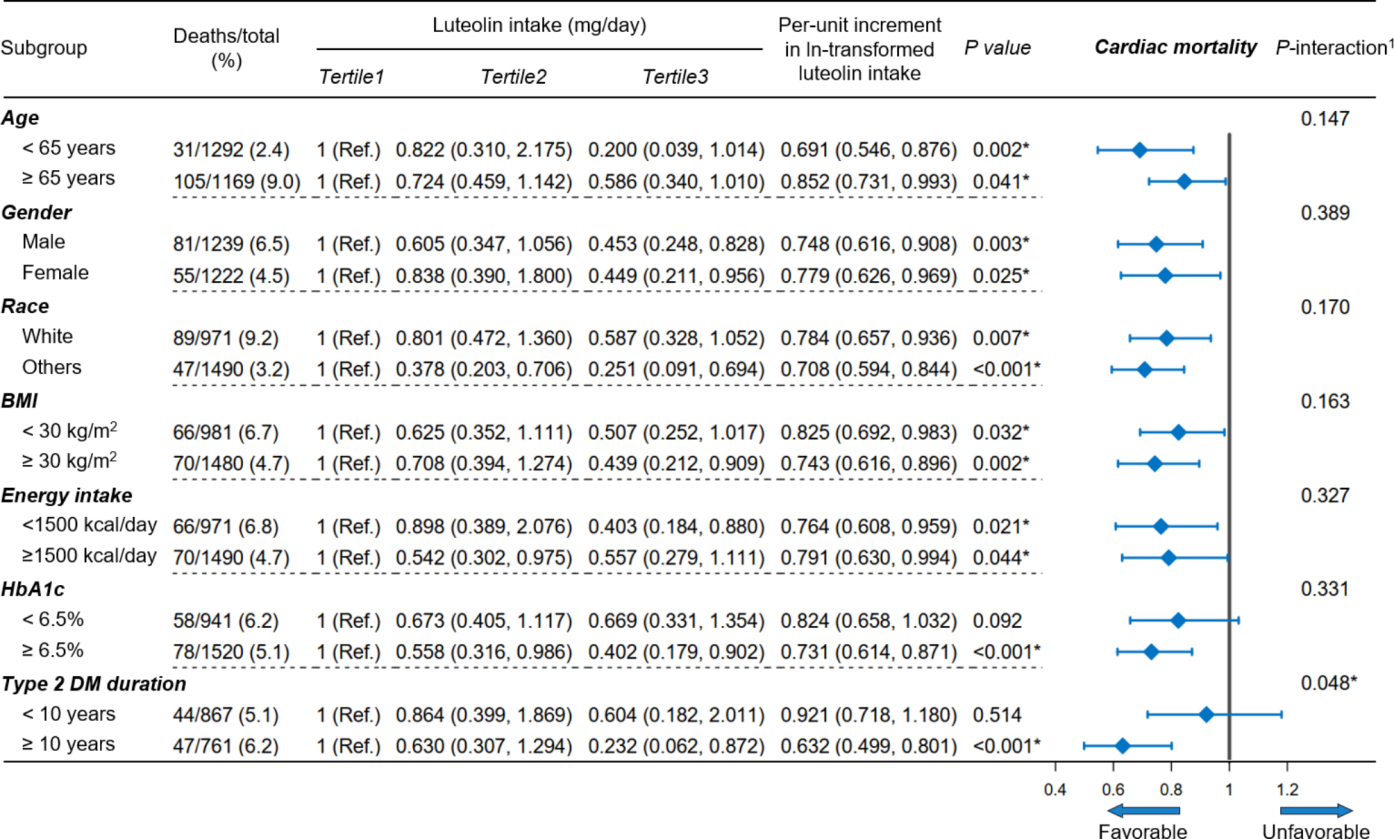
Subgroup analyses of luteolin intake with cardiac mortality among patients with type 2 diabetes mellitus HR (95% CI) was assessed by Cox proportional hazards model. The model was adjusted for covariates including age, race, gender, body mass index, income-poverty ratio, smoking, drinking, energy intake, physical activity, hypertension, hyperlipidemia, HbA1c level, and use of oral anti-diabetic agents or insulin (except the stratified variable itself). Due to not being informed of their diabetes, 833 patients were unable to determine the diabetes duration and were excluded from the stratified analysis of type 2 DM duration ^1^ The interaction between luteolin intake (continuous) and the stratified variable was assessed by the Wald test *P < 0.05

In Fig. 3 and Figure S3, subgroup analyses revealed a consistent result when patients were stratified by age (< 65, ≥ 65 years), gender (male, female), race (non-Hispanic white, others), BMI (< 30, ≥ 30 kg/m^2^), energy intake (< 1500, ≥ 1500 kcal/day), HbA1c level (< 6.5, ≥ 6.5%), and T2DM duration (< 10, ≥ 10 years). For cardiac mortality (Fig. 3), patients with T2DM duration ≥ 10 years were more likely to get benefits from dietary luteolin intake increment compared to those with T2DM duration < 10 years (*P* for interaction = 0.048). Moreover, patients with HbA1c ≥ 6.5% (adjusted-HR = 0.731, 95% CI = 0.614, 0.871) had a more remarkable cardiac mortality reduction compared to those with HbA1c < 6.5% (adjusted-HR = 0.824, 95% CI = 0.658, 1.032).

Several sensitivity analyses confirmed the robustness of our findings. First, when additionally adjusting for remaining flavonoids in the model, luteolin intake increment remained independently reduced all-cause and cardiac mortality (Table S5). Second, after excluding potential outliers of luteolin intake (excluding 273 samples outside the 5th and 95th percentiles), the inverse dose-response association between luteolin intake and mortality risk remained (Figure S2). Third, except for cardiac death, there was no significant association between luteolin intake increment and cause-specific death, including malignant neoplasms death (adjusted-HR = 1.039; 95%CI = 0.879, 1.222), diabetes mellitus death (adjusted-HR = 1.112; 95%CI = 0.918, 1.348), and cerebrovascular diseases death (adjusted-HR = 0.863; 95%CI = 0.646, 1.152) (Table S6). Finally, a cross-sectional investigation indicated that luteolin intake increment was related to a lower CRP level at baseline (adjusted-β =-0.332; 95% CI =-0.541, -0.122), but not associated with other cardiometabolic risk factors including HbA1c, fasting blood glucose, HOMA-IR, HOMA-IS, HOMA-β, systolic blood pressure, diastolic blood pressure, and LDL-C (Table S7).

## Discussion

The current study prospectively examined the association of luteolin intake with all-cause and cardiac mortality among 2,461 patients with T2DM. During the follow-up period (median, 8.7 years), per-unit increment of luteolin intake (Ln-transformed) contributed to a 7.0% reduction in all-cause mortality (*P* = 0.024) and a 22.6% reduction in cardiac mortality (*P* = 0.001). This association was independent of other flavonoid intakes and consistent across the stratified population. The anti-inflammatory effect of luteolin was also identified at baseline, which may account for the remarkable prognostic improvement.

Flavonoids are a group of polyphenolic compounds produced in plants, and more than 10,000 flavonoids have been isolated and identified [30]. Among US adults, the average flavonoids intake from the daily diet is about 200–250 mg/day, including flavan-3-ols (80%), flavonols (8%), flavanones (6%), anthocyanidins (5%), isoflavones (< 1%), and flavones (< 1%) [31]. Luteolin (3,4,5,7-tetrahydroxy flavone) is a kind of flavones that has been commercially developed as a dietary supplement and cosmetic additive due to its safety properties and multiple biological effects, including anti-diabetic [11], cardiovascular protective [12], anti-inflammatory [13], and anti-cancer [14] effects.

The potent anti-diabetic effect of luteolin has been demonstrated by various animal studies. In the insulin-resistant mouse, Shao et al. found that oral administration of luteolin can reverse glucose intolerance and improve insulin sensitivity [15]. In streptozotocin-induced diabetic nephropathy rats, Xiong et al. found that luteolin administration (80 mg/kg daily for 8 weeks) protected the renal filtration and inhibited glomerulosclerosis and thus delaying the progression of diabetic nephropathy [16]. In diabetic encephalopathy rats, Ren et al. found that luteolin administration improves learning and memory function by inhibiting hyperglycemia-mediated apoptosis in hippocampi [17]. In diabetic rats with chronic wounds, a histopathological study proved that the application of luteolin ointment (0.5% w/w) was effective in accelerating wound healing [18]. Consistently, for the first time, this population-based study confirmed that dietary luteolin intake increment contributed to a risk reduction in both all-cause and cardiac mortality among patients with T2DM. Therefore, it is recommended that T2DM patients consume more luteolin-rich food in their daily diet. To consume more luteolin-rich foods may be a promising dietary intervention strategy for T2DM. Compared to using anti-diabetic drugs, dietary intervention could be safer and can be more easily accepted by patients. Besides, given its anti-diabetic effects, luteolin-rich dietary strategy is also optional for pre-diabetes patients who do not require anti-diabetic drugs.

It is well known that T2DM patients shared a substantially higher cardiovascular risk [32, 33]. In the sensitivity analysis, except for cardiac mortality, there was no significant association of luteolin intake increment with the leading cause-specific mortality (malignant neoplasms, diabetes mellitus, and cerebrovascular diseases). The prognostic improvement of T2DM could be mainly due to cardiac mortality reduction. Consistently, previous animal studies have also identified several therapeutic effects of luteolin on cardiac-related diseases. First, in myocardial ischemia/reperfusion injury, luteolin alleviates cardiomyocyte apoptosis and blocks oxidative stress, thus promoting cytoprotection and reducing ischemia/reperfusion injury [19]. Second, in heart failure, luteolin improves heart function by enhancing contractility, upregulating autophagy, and preventing cardiac fibrosis [20]. Third, in coronary artery disease, luteolin reverses atherosclerosis by ameliorating oxidative damage, decreasing vascular inflammation, and inhibiting the proliferation and migration of vascular smooth muscle cells [21]. In addition, some epidemiological evidence also supports the primary protection of the flavonoid intake increment in reducing mortality risk from coronary heart disease among the disease-free elderly [34].

There are still several findings in our study that are worth noting. First, the anti-inflammatory effect of luteolin may account for cardiac-specific prognosis improvement. Low-grade inflammation is a common feature in subjects with both T2DM and cardiac disease [35]. The potent anti-inflammatory property of luteolin has been well established by previous studies [36, 37]. Consistently, in the sensitivity analysis, we identified the anti-inflammatory property and found that luteolin intake increment was associated with a lower baseline CRP level (β =-0.332, 95% CI =-0.541, -0.122). However, we did not find a significant association between luteolin intake and other cardiometabolic risk factors, including HbA1c, fasting blood glucose, HOMA-IR, HOMA-IS, HOMA-β, arterial blood pressure, and LDL-C. Although some of these have been previously reported. This discrepancy may be due to several reasons. First, the pleiotropic effects of luteolin were primarily confirmed in preclinical animal studies, which may not be generalized to population-based studies. In addition, the dose of luteolin intake in our study was low, which only came from the general diet rather than additional supplements. These low-dose intakes may not reach the threshold of the pleiotropic effect.

For another noteworthy finding, the impact of luteolin intake on cardiac mortality may vary depending on T2DM duration and blood glucose levels. For cardiac mortality, we found that patients with T2DM duration ≥ 10 years were more likely to get benefits from dietary luteolin intake increment compared to those with T2DM duration < 10 years (*P* for interaction = 0.048, Fig. 3). Moreover, patients with HbA1c ≥ 6.5% had a more remarkable cardiac mortality reduction compared to those with HbA1c < 6.5%. The anti-inflammatory mechanism of luteolin may account for this discrepancy. First, low-grade inflammation status is a chronic process that gradually promotes the development of cardiac disease [38]. Therefore, with the T2DM duration increasing, the benefit of luteolin intake then may become more pronounced. Second, patients with uncontrolled blood glucose tend to have a higher level of inflammation [39, 40]. Thus, patients with HbA1c ≥ 6.5% were more likely to benefit from the anti-inflammatory effect of luteolin.

This study still has several limitations. First, the dose of luteolin intake was only estimated at baseline, which might not accurately represent the luteolin intake during follow-up. Second, NHANES took the representative US civilians. In the United States, the western dietary pattern is predominant, which determines the dietary composition of luteolin and other nutrients. Therefore, our findings may not generalize to populations with non-western dietary patterns. Third, some T2DM patients were determined based on questionnaire data and medication data, which may be subject to self-report bias. Fourth, to remove T1DM from all diabetics, we excluded patients who were informed of their diabetes at age < 30 years (n = 145). This may introduce sample selection bias due to considering only the epidemiological characteristics of T1DM. Fifth, distinguishing luteolin from nutrients in foods (especially other flavonoids) remains difficult, although potential dietary confounding factors (energy and various flavonoids) have been adjusted in analyses. Finally, because of the nature of observational studies, we cannot draw a causal inference, and residual or unknown confounding factors may still exist.

## Conclusion

This study confirmed that the dietary luteolin intake increment reduced all-cause mortality (especially cardiac mortality) in patients with T2DM. To avoid premature cardiac complications, it is recommended that T2DM patients consume more luteolin-rich foods in their daily diet. However, further studies are needed to determine whether additional luteolin intake from supplementations is an optional strategy for the primary prevention of cardiac diseases among T2DM patients.

## Electronic supplementary material

Below is the link to the electronic supplementary material.

Supplementary Material 1

## Data Availability

The data presented in this study are openly available in https://wwwn.cdc.gov/nchs/nhanes.

## Abbreviations

T2DM: type 2 diabetes mellitus
NHANES: National Health and Nutrition Examination Survey
MEC: mobile examination center
FNDDS: Food and Nutrient Database for Dietary Studies
ICD-10: International Classification of Diseases, Tenth Revision
BMI: body mass index
LDL-C: low-density lipoprotein cholesterol
HOMA: homeostasis model assessment-insulin resistance
CRP: C-reactive protein
EPV: events per variable
