# Supplementary material for: Luteolin intake is negatively associated with all-cause and cardiac mortality among patients with type 2 diabetes mellitus
Source: Diabetol Metab Syndr. 2023 Mar 25;15:59. doi: 10.1186/s13098-023-01026-9 (PMC10039598; doi:10.1186/s13098-023-01026-9)
Supplement: Supplementary file 1 — Supplementary Material 1 [file 13098_2023_1026_MOESM1_ESM.docx]

**Supplementary Materials**

**Supplementary Figures**

**Figure S1.** Flowchart of the patient selection

**Figure S2.** The dose-response association of luteolin intake (excluding potential outliers) with all-cause mortality (A) and cardiac mortality (B) among patients with type 2 diabetes mellitus

**Figure S3.** Subgroup analyses of luteolin intake with all-cause mortality among patients with type 2 diabetes mellitus

**Supplementary Tables**

**Table S1.** Dietary dictionary of luteolin in foods and beverages (≥1mg/100mg)

**Table S2.** Definition and proportion of cause of death

**Table S3.** Classification of flavonoids

**Table S4.** RR (95% CI) for all-cause and cardiac mortality based on luteolin intake among patients with type 2 diabetes mellitus

**Table S5.** HR (95% CI) for all-cause and cardiac mortality based on luteolin intake (additionally adjusting for multiple flavonoids) among patients with type 2 diabetes mellitus

**Table S6.** HR (95% CI) for cause-specific death based on luteolin intake among patients with type 2 diabetes mellitus

**Table S7.** Association between luteolin intake and baseline cardiometabolic risk factors among patients with type 2 diabetes mellitus

**Figure S1.** Flowchart of the patient selection


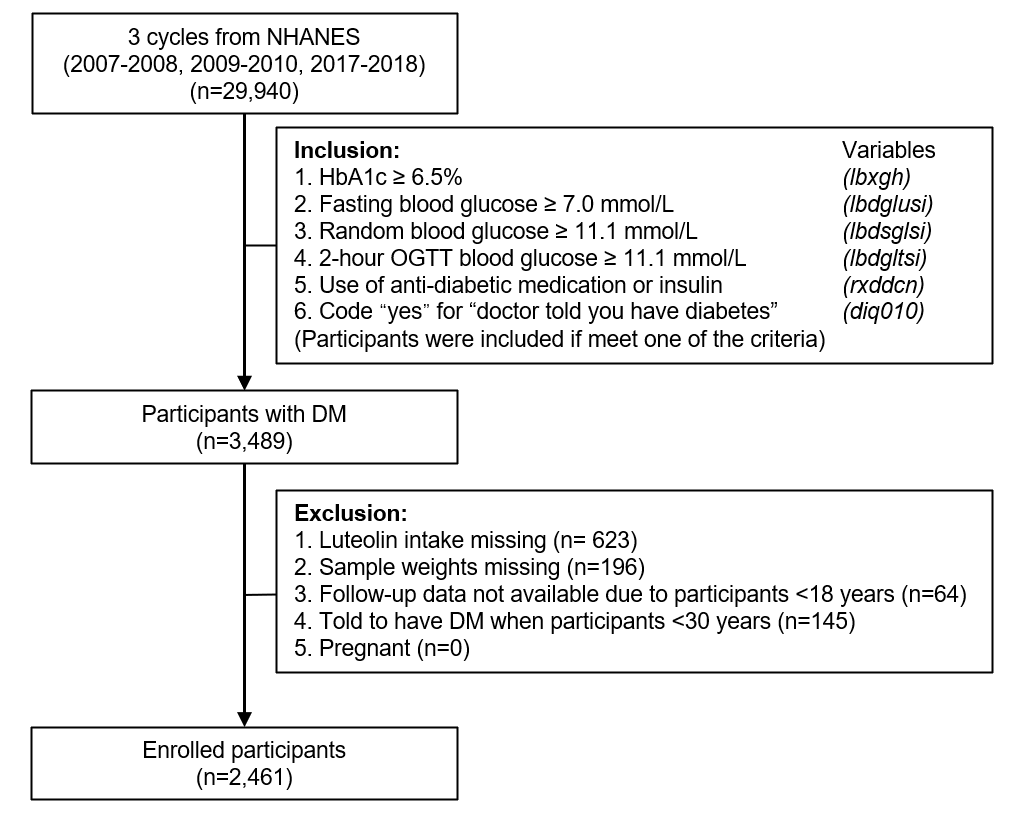


**Figure S2.** The dose-response association of luteolin intake (excluding potential outliers) with all-cause mortality (A) and cardiac mortality (B) among patients with type 2 diabetes mellitus


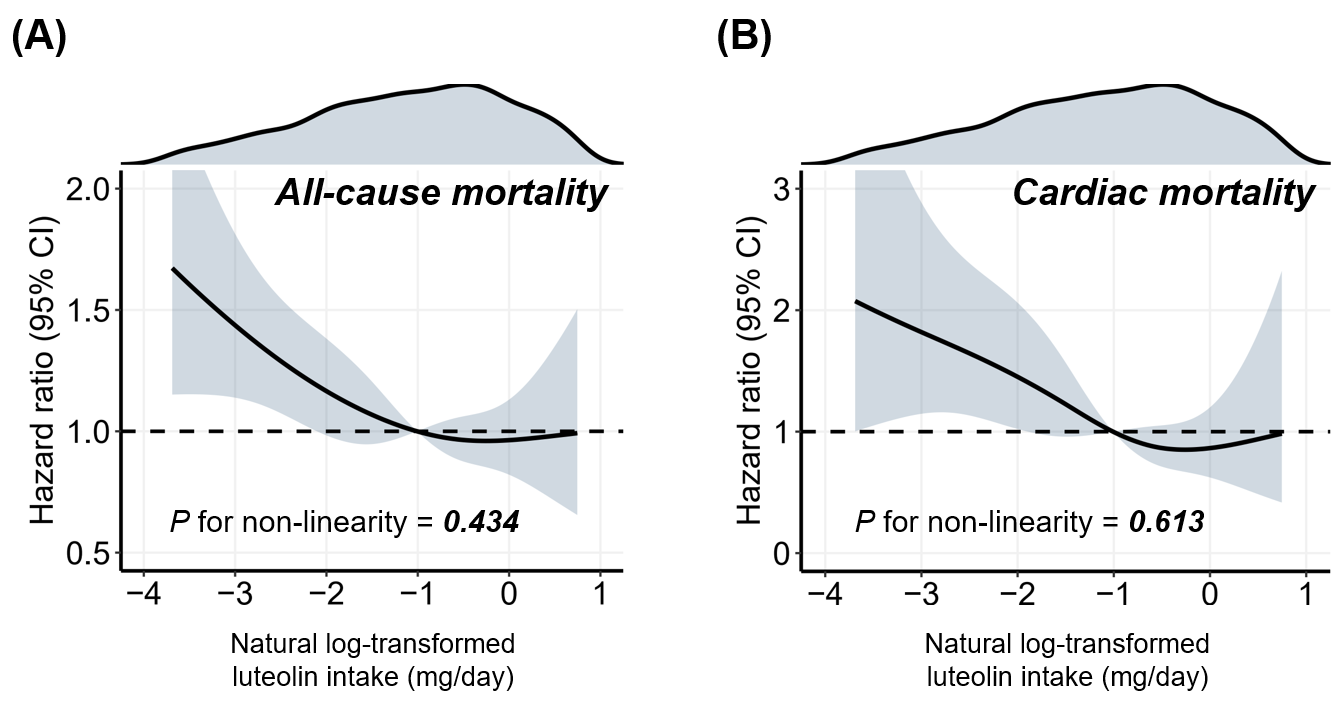


To avoid the potential effect of outliers, we excluded patients with luteolin intake outside the 5th and 95th percentile (excluding 273 samples). Then, a restricted cubic spline model was employed to visualize the relationship between luteolin intake (0.020-2.17mg/day) and mortality risk with four knots determined at the 5th, 35th, 65th, and 95th percentiles. For more details on the spline model, refer to Figure 3.

**Figure S3.** Subgroup analyses of luteolin intake with all-cause mortality among patients with type 2 diabetes mellitus


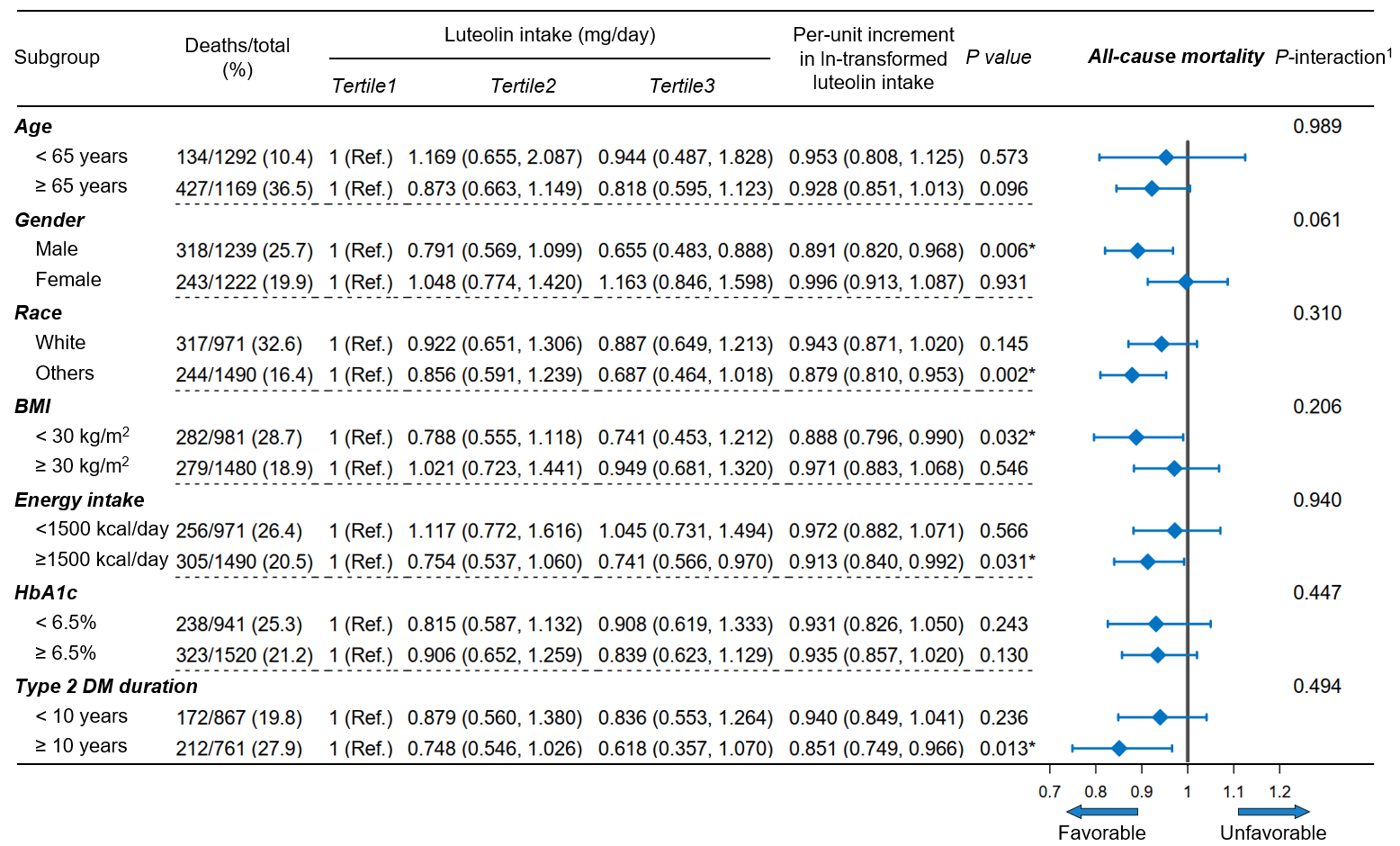


HR (95% CI) was assessed by Cox proportional hazards model. The model was adjusted for covariates including age, race, gender, body mass index, income-poverty ratio, smoking, drinking, energy intake, physical activity, hypertension, hyperlipidemia, HbA1c level, and use of oral anti-diabetic agents or insulin (except the stratified variable itself). Due to not being informed of their diabetes, 833 patients were unable to determine the diabetes duration and were excluded from the stratified analysis of type 2 DM duration.

^1^ The interaction between luteolin intake (continuous) and the stratified variable was assessed by the Wald test.

**P*<0.05

**Table S1.** Dietary dictionary of luteolin in foods and beverages (≥1mg/100mg)

| Rank | Luteolin Value  (mg/100g) |  | Food Description |
| --- | --- | --- | --- |
| 1 | 37.98 |  | Radicchio, raw |
| 2 | 6.32 |  | Pepper, banana, raw |
| 3 | 5.53 |  | Pimiento |
| 4 | 5.29 |  | Pepper, hot chili, raw |
| 5 | 4.74 |  | Pepper, hot, pickled |
| 6 | 4.71 |  | Pepper, poblano, raw |
| 7 | 4.71 |  | Pepper, sweet, green, raw |
| 8 | 4.53 |  | Hot peppers, cooked |
| 9 | 4.3 |  | Green pepper, cooked, as ingredient |
| 10 | 4.14 |  | Pepper, Serrano, raw |
| 11 | 4.04 |  | Peppers, green, cooked |
| 12 | 3.28 |  | Pepper, raw, NFS |
| 13 | 3.28 |  | Pepper, for use on a sandwich |
| 14 | 2.82 |  | Okra, fresh, cooked, no added fat |
| 15 | 2.8 |  | Olives, NFS |
| 16 | 2.8 |  | Olives, black |
| 17 | 2.75 |  | Stuffed pepper, with meat |
| 18 | 2.74 |  | Okra, NS as to form, cooked |
| 19 | 2.74 |  | Okra, fresh, cooked, fat added |
| 20 | 2.74 |  | Luffa, cooked |
| 21 | 2.73 |  | Vegetable mixture, dried |
| 22 | 2.58 |  | Stuffed pepper, with rice, meatless |
| 23 | 2.5 |  | Horseradish |
| 24 | 2.5 |  | Wasabi paste |
| 25 | 2.44 |  | Stuffed green pepper, Puerto Rican style |
| 26 | 2.37 |  | Okra, frozen, cooked, no added fat |
| 27 | 2.33 |  | Blueberries, dried |
| 28 | 2.31 |  | Peppers, pickled |
| 29 | 2.3 |  | Okra, frozen, cooked, fat added |
| 30 | 2.18 |  | Okra, pickled |
| 31 | 2.03 |  | Lime, raw |
| 32 | 1.97 |  | Topping from vegetable pizza |
| 33 | 1.9 |  | Lemon, raw |
| 34 | 1.89 |  | Puerto Rican seasoning without ham and tomato sauce |
| 35 | 1.83 |  | Lemon juice, 100%, canned or bottled |
| 36 | 1.81 |  | Vada, fried dumpling |
| 37 | 1.8 |  | Blueberries, frozen |
| 38 | 1.78 |  | Winter squash, cooked, no added fat |
| 39 | 1.74 |  | Chiles rellenos, cheese-filled |
| 40 | 1.72 |  | Winter squash, cooked, fat added |
| 41 | 1.68 |  | Chiles rellenos, filled with meat and cheese |
| 42 | 1.63 |  | Winter squash, raw |
| 43 | 1.61 |  | Stuffed pepper, with rice and meat |
| 44 | 1.6 |  | Pumpkin, canned, cooked |
| 45 | 1.59 |  | Puerto Rican seasoning with ham and tomato sauce |
| 46 | 1.59 |  | Olive tapenade |
| 47 | 1.54 |  | Pepper steak |
| 48 | 1.52 |  | Peppers and onions, cooked, no added fat |
| 49 | 1.48 |  | Peppers and onions, cooked, fat added |
| 50 | 1.45 |  | Puerto Rican seasoning with ham |
| 51 | 1.44 |  | Pumpkin, cooked |
| 52 | 1.44 |  | Calabaza, cooked |
| 53 | 1.37 |  | Squash, winter, souffle |
| 54 | 1.3 |  | Kohlrabi, raw |
| 55 | 1.25 |  | Bean chips |
| 56 | 1.23 |  | Spaghetti squash, cooked |
| 57 | 1.14 |  | Cactus, raw |
| 58 | 1.11 |  | Lentils, from dried, no added fat |
| 59 | 1.11 |  | Kohlrabi, cooked |
| 60 | 1.11 |  | Olives, stuffed |
| 61 | 1.1 |  | Sausage and peppers, no sauce |
| 62 | 1.09 |  | Parsley, raw |
| 63 | 1.07 |  | Summer squash, yellow or green, frozen, cooked, no added fat |
| 64 | 1.05 |  | Celery, raw |
| 65 | 1.05 |  | Celery juice |
| 66 | 1.04 |  | Lentils, NFS |
| 67 | 1.04 |  | Lentils, from dried, fat added |
| 68 | 1.04 |  | Lentils, from canned |
| 69 | 1.04 |  | Summer squash, yellow, raw |
| 70 | 1.04 |  | Summer squash, yellow or green, frozen, cooked, fat added, NS as to fat type |
| 71 | 1.04 |  | Summer squash, yellow or green, frozen, cooked with oil |
| 72 | 1.04 |  | Summer squash, yellow or green, frozen, cooked with butter or margarine |
| 73 | 1.02 |  | Summer squash, yellow or green, fresh, cooked, no added fat |
| 74 | 1.02 |  | Summer squash, cooked, as ingredient |
| 75 | 1.01 |  | Summer squash, green, raw |

**Table S2.** Definition and proportion of cause of death

| Cause of death (ICD-10 codes) | Count | Proportion (%) |
| --- | --- | --- |
| All other causes (residual) | 143 | 25.5 |
| Diseases of heart (I00-I09, I11, I13, I20-I51) | 136 | 24.2 |
| Malignant neoplasms (C00-C97) | 124 | 22.1 |
| Diabetes mellitus (E10-E14) | 44 | 7.8 |
| Cerebrovascular diseases (I60-I69) | 38 | 6.8 |
| Chronic lower respiratory diseases (J40-J47) | 29 | 5.2 |
| Influenza and pneumonia (J09-J18) | 16 | 2.9 |
| Alzheimer's disease (G30) | 12 | 2.1 |
| Nephritis, nephrotic syndrome and nephrosis (N00-N07, N17-N19, N25-N27) | 12 | 2.1 |
| Accidents (unintentional injuries) (V01-X59, Y85-Y86) | 7 | 1.3 |
| Total | 561 | 100 |

The survival outcomes were as of December 31, 2019. The causes of death were defined by the International Classification of Diseases, Tenth Revision (ICD-10).

**Table S3.** Classification of flavonoids

| Flavonoid class | Flavonoid | |
| --- | --- | --- |
| Flavones | 1 | ***Luteolin*** |
|  | 2 | Apigenin |
| Anthocyanidins | 3 | Cyanidin |
|  | 4 | Delphinidin |
|  | 5 | Malvidin |
|  | 6 | Pelargonidin |
|  | 7 | Peonidin |
|  | 8 | Petunidin |
| Flavan-3-ols | 9 | (-)-Epicatechin |
|  | 10 | (-)-Epicatechin 3-gallate |
|  | 11 | (-)-Epigallocatechin |
|  | 12 | (-)-Epigallocatechin 3-gallate |
|  | 13 | (+)-Catechin |
|  | 14 | (+)-Gallocatechin |
|  | 15 | Theaflavin |
|  | 16 | Theaflavin-3,3'-digallate |
|  | 17 | Theaflavin-3'-gallate |
|  | 18 | Theaflavin-3-gallate |
|  | 19 | Thearubigins |
| Flavanones | 20 | Eriodictyol |
|  | 21 | Hesperetin |
|  | 22 | Naringenin |
| Flavonols | 23 | Isorhamnetin |
|  | 24 | Kaempferol |
|  | 25 | Myricetin |
|  | 26 | Quercetin |
| Isoflavones | 27 | Daidzein |
|  | 28 | Genistein |
|  | 29 | Glycitein |

The flavonoid database was part of the Food and Nutrient Database for Dietary Studies (FNDDS) and was managed by the United States Department of Agriculture (USDA). The flavonoid database calculated the amounts of 29 flavonoids (6 flavonoid classes) in all foods and beverages corresponding to the NHANES dietary data.

**Table S4.** RR (95% CI) for all-cause and cardiac mortality based on luteolin intake among patients with type 2 diabetes mellitus

| Characteristic | Luteolin intake (mg/day) | | |
| --- | --- | --- | --- |
|  | Tertile 1: 0.005-0.190 | Tertile 2: 0.190-0.635 | Tertile 3: 0.635-9.870 |
| All-cause mortality |  |  |  |
| No. deaths/total (%) | 228/877 (26) | 205/866 (23.7) | 158/863 (18.3) |
| RR | 1 (reference) | 0.910 (0.770, 1.076) | 0.689 (0.573, 0.829) |
| Cardiac mortality |  |  |  |
| No. deaths/total (%) | 64/877 (7.3) | 49/866 (5.7) | 32/863 (3.7) |
| RR | 1 (reference) | 0.760 (0.524, 1.102) | 0.481 (0.313,0.740) |

RR indicates relative risk.

**Table S5.** HR (95% CI) for all-cause and cardiac mortality based on luteolin intake (additionally adjusting for multiple flavonoids) among patients with type 2 diabetes mellitus

| Characteristic | Luteolin intake (mg/day) | | | Per-unit increment  of luteolin intake  (Ln-transformed) | *P* value |
| --- | --- | --- | --- | --- | --- |
|  | Tertile 1 | Tertile 2 | Tertile 3 |  |  |
|  | [0.005, 0.195) | [0.195, 0.640) | [0.640, 9.870] |  |  |
| **All-cause mortality** | | | | | |
| No. deaths/total (%) | 221/841 (26.3) | 192/803 (23.9) | 148/817 (18.1) | 561/2461 (22.8) |  |
| Model 1 (Total Anthocyanidins) | 1 (Ref.) | 0.871 (0.672, 1.130) | 0.833 (0.651, 1.065) | 0.923 (0.859, 0.992) | 0.030* |
| Model 2 (Total Flavan-3-ols) | 1 (Ref.) | 0.872 (0.675, 1.127) | 0.844 (0.674, 1.058) | 0.926 (0.865, 0.991) | 0.026* |
| Model 3 (Total Flavanones) | 1 (Ref.) | 0.852 (0.653, 1.111) | 0.823 (0.668, 1.015) | 0.918 (0.862, 0.978) | 0.008* |
| Model 4 (Total Flavonols) | 1 (Ref.) | 0.897 (0.692, 1.162) | 0.869 (0.672, 1.124) | 0.933 (0.864, 1.008) | 0.079 |
| Model 5 (Total Isoflavones) | 1 (Ref.) | 0.877 (0.673, 1.143) | 0.847 (0.68, 1.056) | 0.927 (0.868, 0.989) | 0.022* |
| Model 6 (Apigenin) | 1 (Ref.) | 0.917 (0.704, 1.193) | 0.909 (0.731, 1.129) | 0.945 (0.883, 1.012) | 0.105 |
| **Cardiac mortality** | | | | | |
| No. deaths/total (%) | 62/841 (7.4) | 45/803 (5.6) | 29/817 (3.5) | 136/2461 (5.5) |  |
| Model 1 (Total Anthocyanidins) | 1 (Ref.) | 0.667 (0.408, 1.090) | 0.495 (0.282, 0.867) | 0.776 (0.664, 0.906) | 0.001* |
| Model 2 (Total Flavan-3-ols) | 1 (Ref.) | 0.689 (0.418, 1.136) | 0.502 (0.293, 0.861) | 0.782 (0.675, 0.905) | 0.001* |
| Model 3 (Total Flavanones) | 1 (Ref.) | 0.646 (0.396, 1.055) | 0.475 (0.273, 0.827) | 0.768 (0.659, 0.895) | 0.001* |
| Model 4 (Total Flavonols) | 1 (Ref.) | 0.711 (0.430, 1.175) | 0.531 (0.311, 0.908) | 0.791 (0.684, 0.914) | 0.001* |
| Model 5 (Total Isoflavones) | 1 (Ref.) | 0.664 (0.413, 1.066) | 0.488 (0.285, 0.836) | 0.775 (0.669, 0.897) | 0.001* |
| Model 6 (Apigenin) | 1 (Ref.) | 0.653 (0.405, 1.054) | 0.475 (0.265, 0.851) | 0.764 (0.653, 0.894) | 0.001* |

HR (95% CI) was assessed by Cox proportional hazards model. All models were adjusted for age, race, gender, body mass index, income-poverty ratio, smoking, drinking, energy intake, physical activity, hypertension, hyperlipidemia, HbA1c level, and use of oral anti-diabetic agents or insulin. Moreover, we additionally adjusted for the other 5 types of flavonoid intakes (anthocyanidins, flavan-3-ols, flavanones, flavonols, isoflavones) and apigenin intake, respectively (all in continuous). For more details on the classification of flavonoids, see Table S2.

**P*<0.05

**Table S6.** HR (95% CI) for cause-specific death based on luteolin intake among patients with type 2 diabetes mellitus

| Causes of death | Luteolin intake (mg/day) | | | *P*-trend | Per-unit increment  in ln-transformed  luteolin intake | *P* value |
| --- | --- | --- | --- | --- | --- | --- |
|  | Tertile 1  [0.005,0.195) | Tertile 2  [0.195,0.640) | Tertile 3  [0.640,9.870] |  |  |  |
| **Malignant neoplasms** (C00-C97) | | | | | | |
| No. deaths/total (%) | 42/841 (5) | 42/803 (5.2) | 40/817 (4.9) |  | 124/2461 (5.0) |  |
| Model 1 | 1 (reference) | 0.880 (0.524, 1.478) | 0.987 (0.541, 1.804) | 0.927 | 0.945 (0.801, 1.115) | 0.506 |
| Model 2 | 1 (reference) | 1.006 (0.603, 1.679) | 1.300 (0.712, 2.372) | 0.358 | 1.033 (0.876, 1.219) | 0.698 |
| Model 3 | 1 (reference) | 0.983 (0.589, 1.638) | 1.301 (0.722, 2.346) | 0.336 | 1.036 (0.879, 1.222) | 0.671 |
| **Diabetes mellitus** (E10-E14) | | | | | | |
| No. deaths/total (%) | 18/841 (2.1) | 16/803 (2.0) | 10/817 (1.2) |  | 44/2461 (1.8) |  |
| Model 1 | 1 (reference) | 1.292 (0.530, 3.149) | 0.558 (0.186, 1.670) | 0.184 | 0.997 (0.818, 1.216) | 0.978 |
| Model 2 | 1 (reference) | 1.318 (0.561, 3.095) | 0.849 (0.278, 2.589) | 0.675 | 1.138 (0.932, 1.391) | 0.205 |
| Model 3 | 1 (reference) | 1.214 (0.502, 2.935) | 0.794 (0.272, 2.318) | 0.598 | 1.112 (0.918, 1.348) | 0.278 |
| **Cerebrovascular diseases** (I60-I69) | | | | | | |
| No. deaths/total (%) | 15/841 (1.8) | 12/803 (1.5) | 11/817 (1.3) |  | 44/2461 (1.8) |  |
| Model 1 | 1 (reference) | 0.686 (0.297, 1.582) | 0.569 (0.188, 1.719) | 0.372 | 0.852 (0.645, 1.125) | 0.258 |
| Model 2 | 1 (reference) | 0.669 (0.262, 1.709) | 0.548 (0.163, 1.844) | 0.381 | 0.845 (0.629, 1.136) | 0.265 |
| Model 3 | 1 (reference) | 0.633 (0.252, 1.592) | 0.576 (0.174, 1.907) | 0.435 | 0.863 (0.646, 1.152) | 0.317 |

HR (95% CI) for specific causes of death was assessed by Cox proportional hazards model. As for the adjusted covariates in models, refer to Table 2. Specific causes of death included malignant neoplasms (ICD-10, C00 to C97), diabetes mellitus (ICD-10, E10 to E14), and cerebrovascular diseases (ICD-10, I60 to I69). Linear trends in hazard ratios across categories were examined by entering the median luteolin intake for each category as a numeric variable.

**Table S7.** Association between luteolin intake and baseline cardiometabolic risk factors among patients with type 2 diabetes mellitus

| Cardiometabolic risk factors  (Outcomes) | Per-unit increment in luteolin intake (Ln-transformed) | | *P* value |
| --- | --- | --- | --- |
|  | Adjusted-beta | 95% Confidence Interval |  |
| HbA1c | 0.007 | (-0.037, 0.051) | 0.766 |
| FBG | 0.071 | (-0.022, 0.163) | 0.135 |
| HOMA-IR^1^ | 0.314 | (-0.154, 0.782) | 0.189 |
| HOMA-IS^2^ | 0.002 | (-0.012, 0.016) | 0.808 |
| HOMA-β^3^ | -121.781 | (-676.045, 432.483) | 0.667 |
| SBP | -0.235 | (-0.806, 0.337) | 0.421 |
| DBP | -0.178 | (-0.523, 0.166) | 0.310 |
| LDL-C | -0.005 | (-0.044, 0.035) | 0.823 |
| CRP | -0.332 | (-0.541, -0.122) | 0.002* |

Beta (95% CI) was assessed by the linear regression model and accounted for age, race, gender, body mass index, income-poverty ratio, smoking, drinking, energy intake, physical activity, hypertension (except for SBP and DBP outcomes), hyperlipidemia (except for LDL outcome), and HbA1c level (except for HbA1c, FBG, HOMA-IR, HOMA-IS, and HOMA-β outcomes). HOMA indicates homeostasis model assessment; IR, insulin resistance; IS, insulin sensitivity; FBG, fasting blood glucose; SBP, systolic blood pressure; DBP, diastolic blood pressure; LDL-C, low-density lipoprotein cholesterol; CRP, C-reactive protein.

^1^ HOMA-IR reflects insulin resistance and is calculated as (fasting glucose, mmol/L) _plasma_ × (fasting insulin, μU/mL) _plasma_ / 22.5.

^2^ HOMA-IS reflects insulin sensitivity and is calculated as 1/HOMA-IR.

^3^ HOMA-β reflects the function of pancreatic β cells and is calculated as 20 × (fasting insulin, μU/mL) _plasma_ / ((fasting glucose, mmol/L) _plasma_ -3.5) (%)

**P*<0.05
